# Early detection of thymidylate synthase resistance in non-small cell lung cancer with FLT-PET imaging

**DOI:** 10.18632/oncotarget.19751

**Published:** 2017-07-31

**Authors:** Xiao Chen, Yizeng Yang, Sharyn Katz

**Affiliations:** ^1^ Department of Radiology, University of Pennsylvania Perelman School of Medicine, Philadelphia, PA, USA; ^2^ Department of Radiology, Institute of Surgery Research, Daping Hospital, Third Military Medical University, Chongqing, China; ^3^ Department of Medicine, University of Pennsylvania Perelman School of Medicine, Philadelphia, PA, USA

**Keywords:** FLT, PET, pemetrexed, lung cancer, flare

## Abstract

**Introduction:**

Inhibition of thymidylate synthase (TS) results in a transient compensatory “flare” in thymidine salvage pathway activity measureable with ^18^F-thymidine (FLT)- positron emission tomography (PET) at 2hrs. of therapy which may predict non-small cell lung cancer (NSCLC) sensitivity to TS inhibition.

**Materials and Methods:**

Resistance to TS inhibition by pemetrexed was induced in NSCLC cell lines H460 and H1299 through TS overexpression. TS overexpression was confirmed with RT-PCR and Western blotting and pemetrexed resistance confirmed with IC_50_ assays. The presence of a pemetrexed-induced thymidine salvage pathway “flare” was then measured using ^3^H-thymidine in both pemetrexed sensitive (H460 and H1299) and resistant (H460R, H1299R, CALU-6, H522, H650, H661, H820, H1838) lines *in vitro*, and validated with FLT-PET *in vivo* using H460 and H460R xenografts.

**Results:**

Overexpression of TS induced pemetrexed resistance with IC_50_ for H460, H1299, H460R and H1299R measured as 0.141 μM, 0.656 μM, 22.842 μM, 213.120 μM, respectively. Thymidine salvage pathway ^3^H-thymidine “flare” was observed following pemetrexed in H460 and H1299 but not H460R, H1299R, CALU-6, H522, H650, H661, H820 or H1838 *in vitro*. Similarly, a FLT “flare” was observed *in vivo* following pemetrexed therapy in H460 but not H460R tumor-bearing xenografts.

**Conclusions:**

Imaging of TS inhibition is predictive of NSCLC sensitivity to pemetrexed.

## INTRODUCTION

Inhibitors of thymidylate synthase (TS), a key enzyme in the *de novo* thymidine synthesis pathway, play a role in the treatment of a number of malignancies including non-small cell lung cancer (NSCLC). Examples of commonly used TS inhibitors in cancer therapy include 5-fluorouracil (5-FU), pemetrexed and capecitabine. Successful inhibition of TS results in a transient compensatory “flare” in activity of the thymidine salvage pathway [1–4], which also sources thymidine to the dividing cell. As a result, this drug-induced compensatory “flare” in thymidine salvage pathway activity is an indicator of successful TS inhibition. This drug-induced change in tumor metabolism can be made visible through ^18^F-thymidine (FLT)-positron emission tomography (PET)[2, 5–7], an analog of thymidine. FLT, first described by Shields in 1998 [8], is an investigational imaging biomarker of the thymidine salvage pathway currently in use for human clinical trials primarily as a validated surrogate marker of tumor proliferation [9–12]. Here we consider FLT-PET imaging as a means of detecting successful TS targeting by pemetrexed, a TS inhibitor currently in use for NSCLC therapy.

In our recent publication [7], we characterized the kinetics of the pemetrexed-induced thymidine salvage pathway “flare” in a xenograft mouse model of human NSCLC and demonstrated that the peak of the pemetrexed-induced thymidine salvage pathway “flare” consistently occurs at 2 hours of exposure to therapy both *in vitro* and *in vivo*. Beyond that 2 hour time point, the pemetrexed-induced “flare” signal decays, dissipating entirely by 24 hours of therapy at which time the anti-proliferative effects of successful therapy have begun to dominate thymidine salvage pathway activity. We also have determined that the pemetrexed-induced “flare” effect is mediated in part by rapid changes in the activity of thymidine kinase 1 (TK1), the key rate-limiting step in the thymidine salvage pathway enzyme and on the mobilization of equilibrative nucleoside transporter 1 (ENT 1) to the cell surface. ENT1 has been shown to be important for facilitation of thymidine, and thymidine analogues, entry into proliferating cells [13–16]. These findings are consistent with published literature also demonstrating that increases in TK1 activity and/or ENT1 mobilization play a significant role in the TS-inhibition mediated thymidine salvage pathway “flare” in activity [2, 3, 17–19].

The question remains whether this TS inhibitor-induced thymidine salvage pathway “flare” in tumor metabolism can serve as a reliable indicator of therapy success with eventual tumor regression. While there is literature describing the phenomenon the TS-inhibition induced “flare” in the salvage pathway activity, there is very little available data examining whether this strategy can be predictive of drug therapy success in cancer with several published papers yielding mixed results [20, 21]. Therefore careful pre-clinical study is warranted to determine whether the pemetrexed-induced “flare” can be predictive of therapy success in NSCLC. Previously, our laboratory has examined the thymidine salvage pathway “flare” imaging strategy in the setting of pemetrexed sensitivity [7]; we now characterize the “flare” in the setting of pemetrexed resistance. TS overexpression is a common and well-described mechanism of cancer resistance to TS inhibitors including NSCLC resistance to pemetrexed [22–25]. In this study we employ TS overexpression to induce resistance to TS inhibition in order to study the pemetrexed-induced “flare” in thymidine salvage pathway activity in the setting of NSCLC drug resistance.

## RESULTS

### Overexpression of TS is associated with resistance to pemetrexed in NSCLC lines *in vitro*

**Figure 1:**
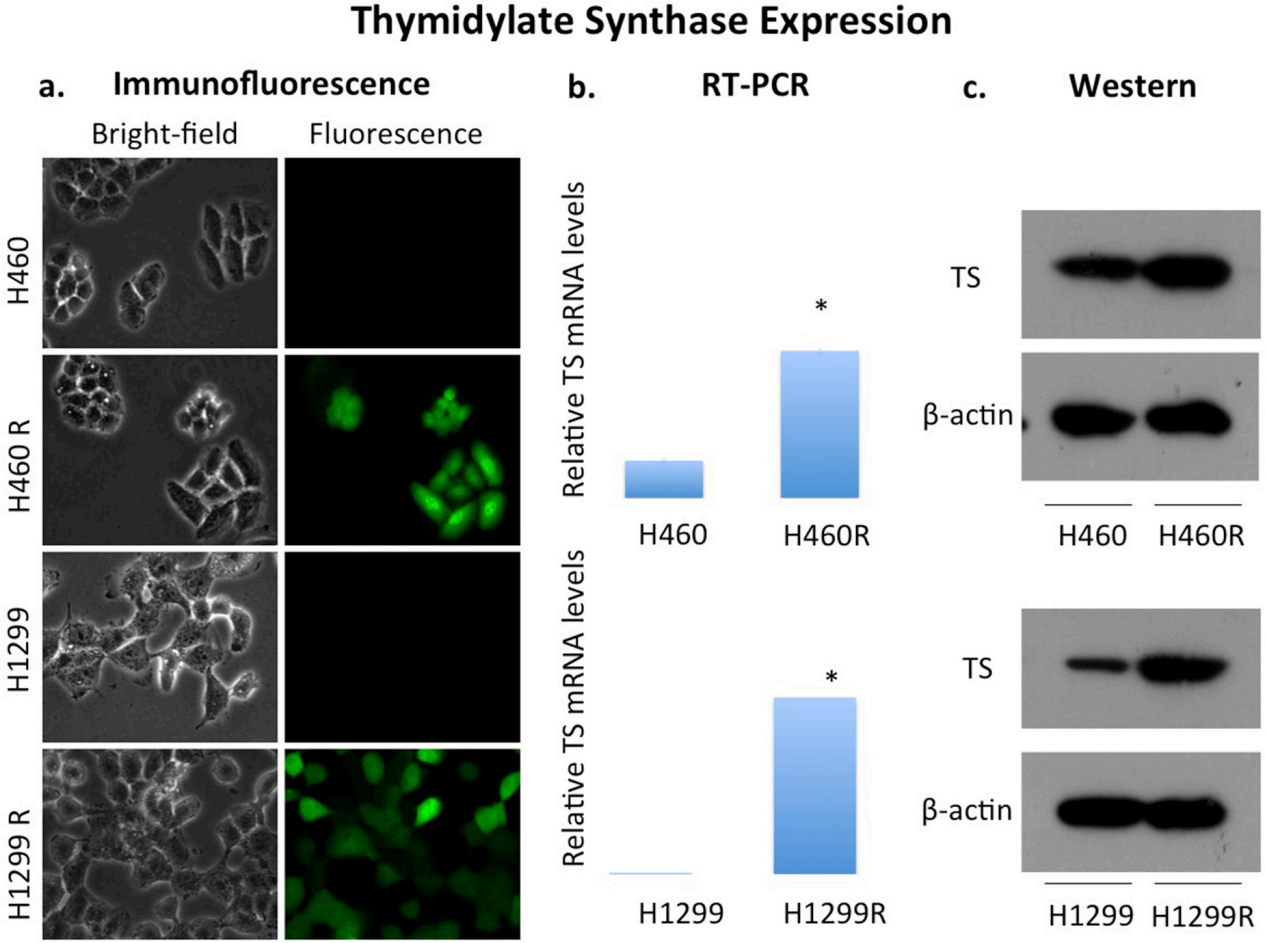
Overexpression of thymidylate synthase in NSCLC cell lines H460 and H1299 Pemetrexed sensitive human NSCLC cell lines H460 and H1299 expressed thymidylate synthase (TS) at low levels at baseline. Overexpression of TS in both cell lines, termed H460R and H1299R, was confirmed with **(a)** detection of GFP in transfected cells by immunofluorescence (TS), **(b)** quantification of TS mRNA by RT-PCR and **(c)** protein expression.

Overexpression of TS in H460 and H1299 cell lines was confirmed *in vitro* using RT-PCR, Western blotting and immunofluorescence (Figure 1 and Supplementary Figure 1). IC_50_ measurements confirmed pemetrexed sensitivity in the wild-type H460 and H1299 cell lines and the development of pemetrexed resistance in the TS overexpressing H460R and H1299R cell lines with IC_50_ measurements of 0.141 μM, 22.8 μM, 0.656 μM, 213 μM for H460, H460R, H1299 and H1299R respectively (Supplementary Figure 2).

In addition, the TS protein expression was also noted to be elevated in six inherently pemetrexed resistant NSCLC cell lines CALU6, H522, H650, H661, H820, H1838 relative to pemetrexed-sensitive cell lines H460 and H1299 (Supplementary Figure 1) with IC50 measurements of (Supplementary Figure 2). The IC50 for CALU6, H522, H650, H661, H820, H1838 were 14.2, 59.6, 238, 85.0, 48.0, 121 μM respectively.

### Overexpression of TS induces resistance to pemetrexed in NSCLC lines H460 and H1299 *in vivo*

Xenografts bearing H460 (16 mice) or H460R (16 mice) were treated as either vehicle only controls (8 mice per cell line) or given combination therapy with pemetrexed and cisplatin (8 mice per cell line) for 2 weeks. Routine external caliper tumor measurements were compared between treated and control groups for each cell line. A significant tumor growth inhibition was observed in the pemetrexed-sensitive H460 chemotherapy treated tumors (74.7±15.1%; p=0.0002) relative to untreated vehicle only controls. There was a slight tumor growth inhibition in the treated pemetrexed-resistant H460R tumors when compared to vehicle only treated controls (47.8±14.4%, p=0.0001) (Supplementary Figure 3).

### Induction of pemetrexed resistance through TS overexpression results in loss of thymidine salvage pathway “flare” *in vitro*

**Figure 2:**
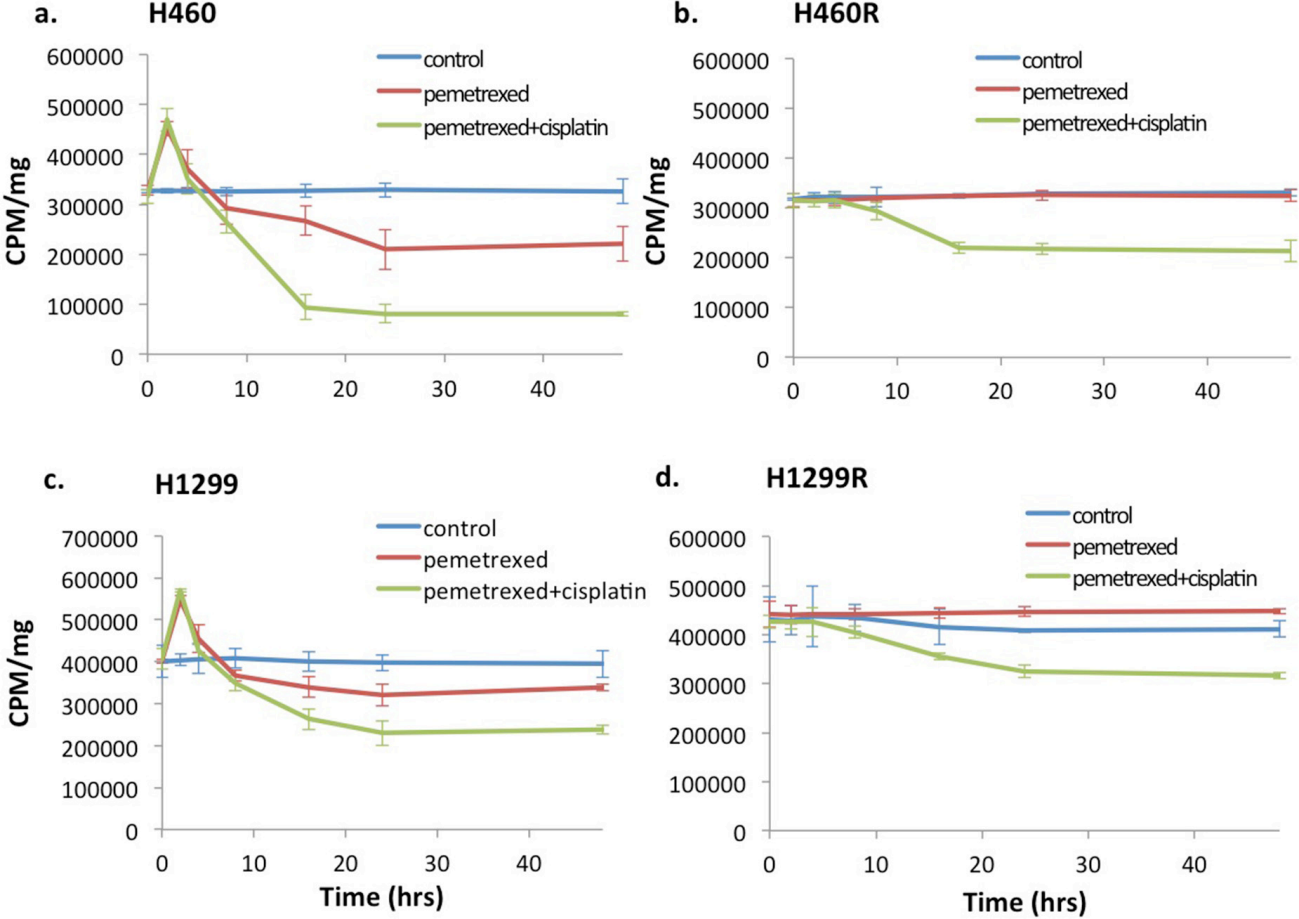
Pemetrexed-resistance conferred by TS overexpression results in loss of the pemetrexed-induced DNA salvage pathway “flare” in NSCLC *in vitro* Pemetrexed-sensitive wild type NSCLC cell lines H460 **(a)** and H1299 **(c)** demonstrated a “flare” in thymidine salvage pathway on 3H-thymidine assay at 2 hrs. of exposure to pemetrexed. The overexpression of TS eliminated the pemetrexed-induced “flare” in thymidine salvage pathway activity in both cell lines, H460R **(b)** and H1299R **(d)**. The exposure to cisplatin did not impact the presence of the pemetrexed-induced thymidine salvage pathway “flare”.

**Figure 3:**
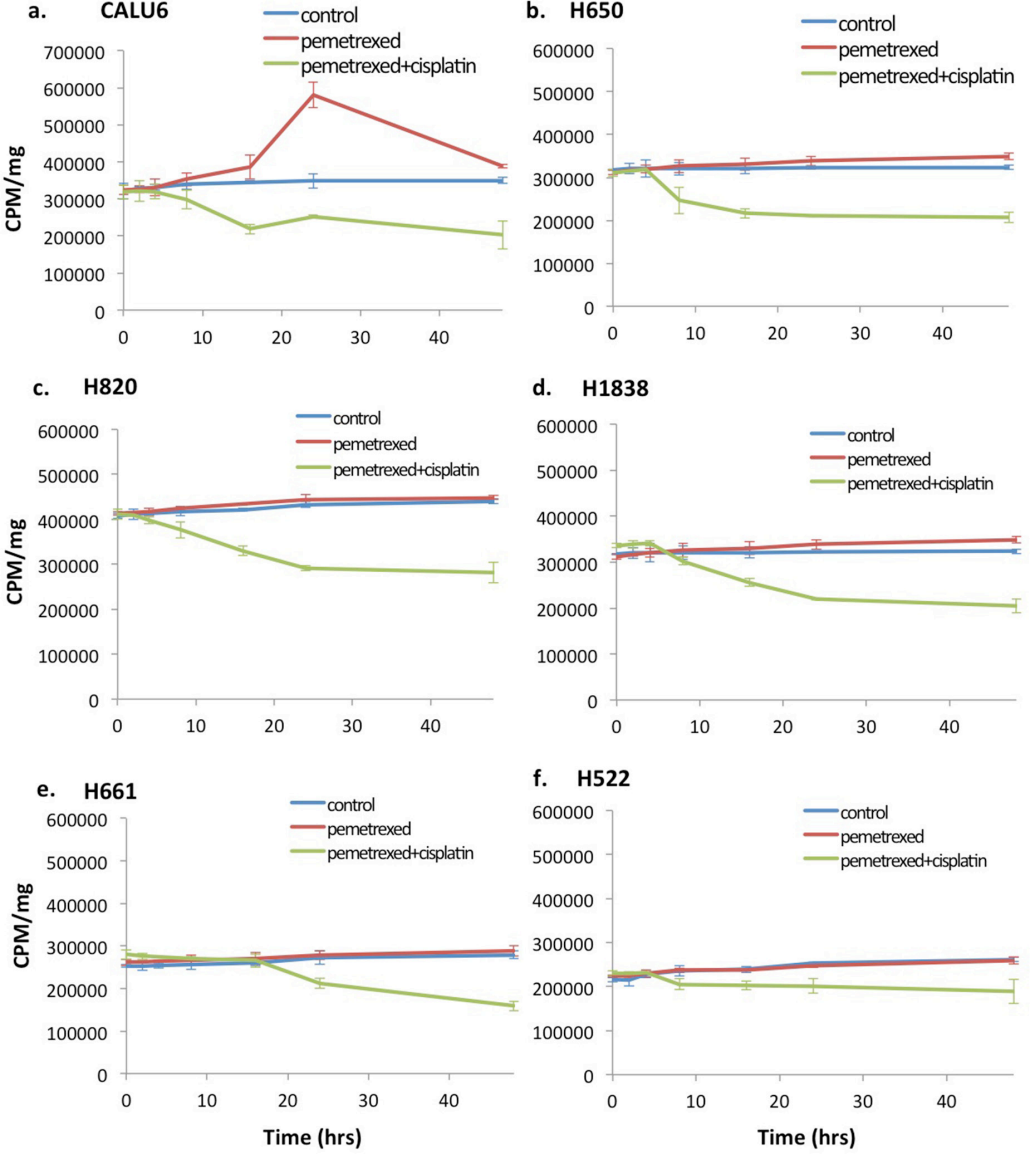
Wild-type resistance to pemetrexed in NSCLC cell lines results in loss of the thymidine salvage pathway induced “flare” at the 2hr time point 3H-thymidine assay was performed on inherently pemetrexed-resistant NSCLC cell lines **(a-f)** following incubation without chemotherapy (culture medium only), pemetrexed (100 nM) or combination therapy with pemetrexed (100 nM) plus cisplatin (10mM). No “flare” in thymidine salvage pathway activity was seen at 2 hrs. of exposure to pemetrexed in any of the 6 pemetrexed-resistant cell lines. One cell line, CALU6 (a), demonstrated a delayed increase in thymidine pathway activity occurring at approximately 24 hrs. of therapy. Exposure to cisplatin showed no impact on the pemetrexed induced “flare”.

Thymidine salvage pathway activity was assayed with ^3^H-thymidine at baseline and following treatment with either pemetrexed, cisplatin or combination of cisplatin and pemetrexed *in vitro*. A “flare” in thymidine salvage pathway activity measured in pemetrexed-sensitive NSCLC lines H460 (43.4±5.11%, p=0.0005) and H1299 (39.5±6.51%, p<0.0001) but not in TS overexpressed pemetrexed-resistant NSCLC cell lines H460R (-2.44±0.95%, p=0.421) and H1299R (-0.60±4.40%, p=0.870) *in vitro* using ^3^H-thymidine assays (Figure 2). In addition, none of the six lines inherently NSCLC resistant NSCLC cells had a pemetrexed-induced thymidine salvage pathway “flare” at 2 hours (CALU6: -1.87±7.20%, p=0.742; H522: 7.63±6.00%, p=0.115; H650: -1.73±3.99%, p=0.454; H661: 3.82±5.13%, p=0.201; H820: -0.03±3.37%, p=0.958; H1838: 3.21±5.20%, p=0.250) (Figure 3). One cell line, CALU6, had a delayed “flare” at 24 hours following start of exposure to pemetrexed therapy (66.9±8.08%, p=0.0005).

### Induction of pemetrexed resistance through TS overexpression results in loss of thymidine salvage “flare” *in vivo*

**Figure 4:**
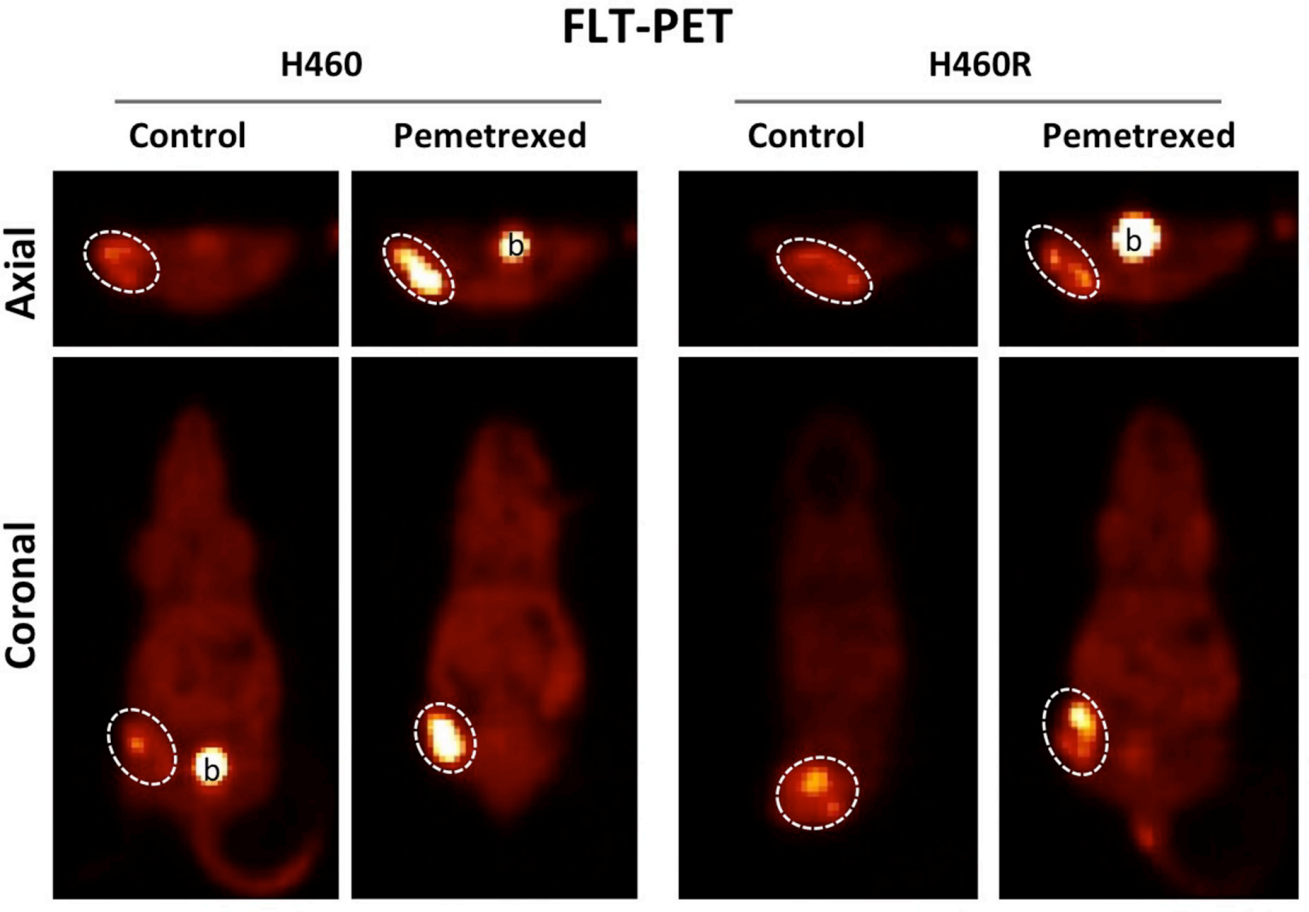
Pemetrexed-resistance conferred by TS overexpression results in loss of the pemetrexed-induced thymidine salvage pathway “flare” in NSCLC *in vivo* FLT-PET imaging was performed on pemetrexed-sensitive H460 and pemetrexed-resistant H460R xenografts the day before and 2 hours after exposure to combination therapy with pemetrexed and cisplatin. A pemetrexed-induced “flare” in thymidine salvage pathway activity is observed in the pemetrexed sensitive H460 xenografts but not the resistant H460R xenografts.

A total of 4 groups of xenografted mice were imaged with FLT-PET at baseline and at 2 hours of therapy and changes in tumor avidity for FLT were measured. These groups were as follows: H460 (8 mice; vehicle only), H460R (8 mice; vehicle only), H460 (8 mice; cisplatin/pemetrexed), H460R (8 mice; cisplatin/pemetrexed). A FLT “flare” in tumor avidity was observed at 2 hours of therapy for pemetrexed-sensitive H460 xenografts (58.6 ±16.1% increase over baseline; p=0.043) but not pemetrexed-resistant H460R xenografts (10.8 ±7.28% increase over baseline; p=0.653) (Figure 4). No FLT “flare” was observed in the vehicle-only controls for H460 (1.44±6.35% increase over baseline; p=0.9582) or H460R (2.34±11.86% increase over baseline ; p=0.9527).

## DISCUSSION

Tumor resistance to pemetrexed, a TS inhibitor used in the treatment of NSCLC, is a common problem in oncologic management. Successful TS inhibition results in a transient “flare” in tumor cellular thymidine salvage pathway activity that is measurable *in vitro* and *in vivo* with FLT-PET imaging. In a previous publication, we described the optimal timing of imaging the TS-inhibition mediated “flare” in the thymidine salvage pathway and detailed mechanisms underlying this effect including increasing of TK1 activity and mobilization of ENT1 to the cell surface [7]. In this study we further examine this imaging technique to determine if the presence of the TS-inhibition induced “flare” in the thymidine salvage pathway is predictive of NSCLC tumor sensitivity to pemetrexed.

Since overexpression of TS is a common mechanism of tumor resistance to TS-inhibition, we chose to study the impact of pemetrexed resistance on the thymidine salvage pathway “flare” by overexpressing TS in two previously sensitive NSCLC cell lines, H460 and H1299. All examined pemetrexed-resistant NSCLC cell lines, those created though overexpression of TS and inherently resistant cell lines, demonstrated elevated levels of TS protein expression relative to the pemetrexed sensitive NSCLC cell lines, H460 and H1299. This is in keeping with published literature demonstrating that TS overexpression is a common mechanism for resistance to pemetrexed therapy in NSCLC [22].

Following overexpression of TS, H460R and H1299R cell lines demonstrated the expected resistance to treatment with pemetrexed as measured by IC_50_ assays *in vitro* and relative tumor growth inhibition *in vivo*. The pemetrexed-resistant H460R xenografts still demonstrated some tumor growth inhibition in response to chemotherapy as a result of sensitivity to cisplatin. Combination therapy with cisplatin and pemetrexed therapy was administered in order to model the regimen typically administered to NSCLC patients. Even so, the absence of thymidine salvage pathway FLT-PET “flare” *in vivo* was able to correctly identify tumors exhibiting a suboptimal tumor response to therapy due to pemetrexed resistance.

Two pemetrexed-sensitive human NSCLC cell lines, H460 and H1299, both exhibit a TS-inhibition mediated thymidine salvage pathway flare at 2 hours of exposure to pemetrexed. With induction of pemetrexed-resistance through TS overexpression, both H460R and H1299R cell lines reveal a loss of the TS-inhibition mediated thymidine salvage pathway “flare” at 2 hours *in vitro* and *in vivo*. This loss of the thymidine salvage pathway “flare” was also observed in a panel of inherently pemetrexed-resistant wild-type NSCLC cell lines. Interesting, one of these resistant cell lines, CALU-6, exhibited a delayed increase in thymidine salvage pathway at 24 hours of exposure to therapy. We hypothesize that this delayed increased in thymidine salvage pathway activity at 24 hours may be on the basis of TS-inhibitor-induced increased expression of TK1 as previously reported [2]. It should be noted that in this study we did not folate restrict the murine diet or administer intravenous thymidine phosphorylase prior to imaging, which is sometimes done to prevent saturation of TS or FLT by high murine endogenous levels of folate or thymidine respectively. As a result, our data may underestimate the magnitude of the pemetrexed induced FLT “flare” in NSCLC.

In summary, here we demonstrate evidence that the presence of a thymidine salvage pathway “flare” at 2 hours may be predictive of tumor response to pemetrexed therapy in a preclinical model of human NSCLC. In this study we demonstrated the absence of a TS-inhibition induced “flare” in thymidine salvage pathway activity in all of the 8 pemetrexed-resistant NSCLC cell lines. The data presented here is complementary to our previous report demonstrating the presence of a TS-inhibition mediated thymidine salvage pathway “flare” at 2 hours in all of the 8 pemetrexed-sensitive human NSCLC cell lines examined. Although there is a published clinical pilot study of the FLT “flare” in NSCLC which did not demonstrate a predictive value of this technique [20], it is plausible that other variables such as concurrent treatment with dexamethasone, known to decrease the expression of thymidylate synthase and dihydrofolate reductase and impact tumor responsiveness to pemetrexed [26], could impact the translation of this technique into the clinic. Careful study is needed to determine if the TS-inhibition mediated FLT “flare” could be of value in the clinical setting. If successful, this technique has the potential of determining NSCLC sensitivity to pemetrexed therapy as early as the day of therapy start.

## MATERIALS AND METHODS

### Chemotherapeutics and imaging radiopharmaceuticals

For *in vitro* studies, pemetrexed (Santa Cruz Biotechnology, Dallas, Texas) and cisplatin (Sigma-Aldrich Corp., St. Louis, MO) were provided in solid form, dissolved in water and stored at -20°C as a 0.2 mM and 1 mM stock, respectively. For *in vivo* use, both human and murine, pemetrexed (ALIMTA; Eli Lilly and Company, Indianapolis, IN) and cisplatin (Teva Pharmaceuticals, PetachTikva Israel) were provided freshly prepared as a 1 mg/ml sterile saline solution by the Abramson Cancer Center Pharmacy. For *in vivo* murine studies, chemotherapeutics were stored at 4°C. [^18^F]FLT was produced on site in the University of Pennsylvania PET Center Cyclotron facility. [^18^F]FLT average specific activity was 5.32 +/- 2.14 Ci/umol, and radiochemical purity >99%.

### Cell lines and culture

All human non-small cell lung cancer cell lines were obtained from American Type Culture Collection (ATCC, Manassas, VA). Both cell lines were grown in RPMI medium containing 10% fetal bovine serum (FBS), 100 IU/mL penicillin, and 100 μg/mL streptomycin in a humidified incubator in 5% CO2 at 37°C. Passage of cell lines was performed at 1:3 dilution after detachment using sterile 0.05% trypsin-EDTA solution.

### IC_50_ calculations

Cultured cell lines were harvested and seeded into a 24-well plate (2X10^4^ cells per well) in RPMI1640 culture medium and incubated for 24 hours at 37°C in a 5% CO_2_ incubator. The culture medium was then replaced with 100 uL of fresh medium containing varying concentrations of pemetrexed (0, 0.01, 0.1, 1, 10, 100 μM) and incubated for 72 hours at 37°C in a 5% CO_2_ incubator. The IC_50_ assay was performed then performed using the MTT Cell Growth Assay Kit (Sigma-Aldrich, St. Louis, MO). Absorbance of the converted dye was measured using a Beckman DU-600 Spectrophotometer (Beckman Coulter Life Sciences, Indianapolis, IN) and data analyzed using the statistical software SPSS 19.0 (IBM, Chicago, USA).

### Mouse tumor xenograft modeling

Prior to *in vivo* animal modeling, approval was obtained by the Institutional Animal Care and Use Committee (IACUC) at the University of Pennsylvania. Human tumor-bearing murine xenografts were then created using two month-old female *nu*/*nu* mice (Crl: NUFoxnlnu, Charles River Laboratory, Wilmington, MA) and fed a conventional murine diet. Each mouse was injected subcutaneously in the flank with a suspension of H460 cells (5×10^6^) in sterile, endotoxin-free 50% Matrigel Matrix (Corning Inc., Corning, NY). When tumors reached a mean volume of approximately 200 mm^3^ (volume= π/6 × length × width × height), animals were randomized into treatment groups.

### ^3^H-thymidine assays

[^3^H]-thymidine (Perkin Elmer NET355001MC, PerkinElmer, Waltham, MA) was utilized for *in vitro* assessment of therapy-induced changes in thymidine salvage pathway activity in cultured human NSCLC cells. [^3^H]-thymidine specific activity was >10Ci(370GBq)/mmol and radiochemical purity >97%. H460 and H1299 cells were seeded (1×10^6^/well) in 6-well plate in RPMI1640 supplemented with 10% FBS and antibiotics, incubated 24 hours in 5% CO_2_ at 37°C. When cell cultures achieved 80% confluence, cells were exposed to treatment with either the vehicle (sterile water), pemetrexed (100 nM), or the combination of pemetrexed (100 nM) and cisplatin (10 μM) in growth media for varying exposure times ranging up to 48 hours. Drug-containing medium was then removed, and the cells were then washed and pulsed with 5 μCi [^3^H]-thymidine/well for 1 hour. The cells were then washed and scraped into plastic vials. Scintillant (10 mL; Research Products International Corp., Mount Prospect, IL) was added to each vial and the radioactivity was counted on a scintillation counter (Beckman Coulter LS6500, Beckman Coulter Life Sciences, Indianapolis, IN).

### Gene overexpression

A full-length cDNA fragment encoding TS was obtained from pDONR223-DTYMK plasmid (Addgene) by polymerase chain reaction with the primers TS-F (5’-ATCCCGGGCCTTGAGCGGCCCGGCGCGGG-3’) and TS-R (5’-CTCCGGAACGAATTCTCACTTCCATAGCTC-3’), and subcloned into the Xbal and EcoR I sites of lentiviral vector pUltra (Addgene). The product was verified by sequencing. Lentivirus was packaged by transfecting into HEK 293T cells with the packaging plasmids pMD2-VSV.G and pCMV-dR8.74. Virus-containing medium was harvested 48 hours and 72 hours after transfection and filtered with 0.45 μm Millex HV filters (EMD Millipore). Lenti-X Concentrator (Clontech) and virus-containing medium (v/v: 1:3) were mixed and incubated at 4°C for 1 hour. The viral particles were concentrated by centrifugation at 1,500 × g for 45 minutes at 4°C. The resulting pellet was then suspended in fresh RPMI 1640 medium and used to infect H460 and H1299 cells.

### Immunoblotting

Therapy-induced changes in the protein expression of key components of the thymidine salvage pathway were assessed using Western blot analysis. H460 and H1299 cells were seeded (1×10^6^ cells/well) in 6-well plate in RPMI1640 supplemented with 10% FBS and antibiotics, incubated 24 hour in 5% CO_2_ at 37°C. When cell cultures achieved 80% confluence, cells were exposed to treatment with either the vehicle (sterile water), pemetrexed (100 nM), or the combination of pemetrexed (100 nM) and cisplatin (10 μM) in growth media for varying exposure times ranging up to 48 hours. Whole cell lysates were then generated using a 1% Nonidet P-40 lysis buffer (Sigma-Aldrich Corp., St. Louis, MO). The suspension was homogenized by passages through a 20-gauge syringe needle and nuclear material removed through centrifugation at 14000 rpm for 15 min at 4°C. Cell lysates were then loaded onto a precast Nupage Bis-Tris Gels (invitrogen, Life Technologies, Corp., Grand Island, NY) for electrophoresis then transferred onto Hybond-P PVDF membranes (Sigma-Aldrich Corp., St. Louis, MO) for Western blot analysis. After blocking membranes with 5% non-fat milk in PBS with 0.1% Tween-20 buffer, PVDF membranes were probed using primary antibodies directed against human TK1 (Cell Signaling Technology, Danvers, MA; 1:5000), human TS (Cell Signaling Technology; 1:4000), or human β-actin (Sigma-Aldrich Corp., St. Louis, MO; 1:10000). Membrane were then washed and incubated (1:3000) with species-specific secondary antibodies, either anti-rabbit or anti-mouse, conjugated to horseradish peroxidase (GE Healthcare Life Science; 1:3000) for 1 hour, the proteins were detected using the Immobilon ECL system (EMD Millipore, Billerica, MA) and quantified using Image J software available through the National Institutes of Health (https://imagej.nih.gov/ij/index.html).

### PET imaging

Baseline FLT-PET scans were performed on the day prior to therapy. Mice were then treated with either the vehicle control (i.p. sterile PBS) or combination therapy with pemetrexed (i.p. 100 mg/kg) and cisplatin (10 mg/kg). A post-therapy FLT-PET was then performed at varying time points following administration of therapy.

For the assessment tumor response to therapy, tumor-bearing animals were treated for a period of two weeks with 100 mg/kg pemetrexed (i.p. daily; days 1-5 and 8-12) and 10 mg/kg cisplatin (i.p. once weekly). During this treatment period, tumor volumes were estimated by external caliper measurements. After therapy/imaging completion, mice were euthanized with carbon dioxide inhalation.

After anesthesia with inhaled isofluorane in O_2_ (3% induction, 1.5% maintenance), mice were injected intravenously with 300-350 μCi of [18F]FLT then allowed to r``ecover from the anesthesia during the 60-min uptake time allowed for radiotracer accumulation. At 60 min post-injection, mice were anesthetized and imaged for a 15-min static acquisition on the small animal PET scanner (A-PET, built in collaboration with Philips Medical Systems) located at the University of Pennsylvania Small Animal Imaging Facility.

## SUPPLEMENTARY MATERIALS FIGURES


