# Supplementary material for: Early detection of thymidylate synthase resistance in non-small cell lung cancer with FLT-PET imaging
Source: Oncotarget. 2017 Jul 31;8(47):82705–13. doi: 10.18632/oncotarget.19751 (PMC5669922; doi:10.18632/oncotarget.19751)
Supplement: Supplementary file 1 [file oncotarget-08-82705-s001.pdf]

Early detection of thymidylate synthase resistance in non-small cell lung cancer with FLT-PET imaging

SUPPLEMENTARY MATERIALS

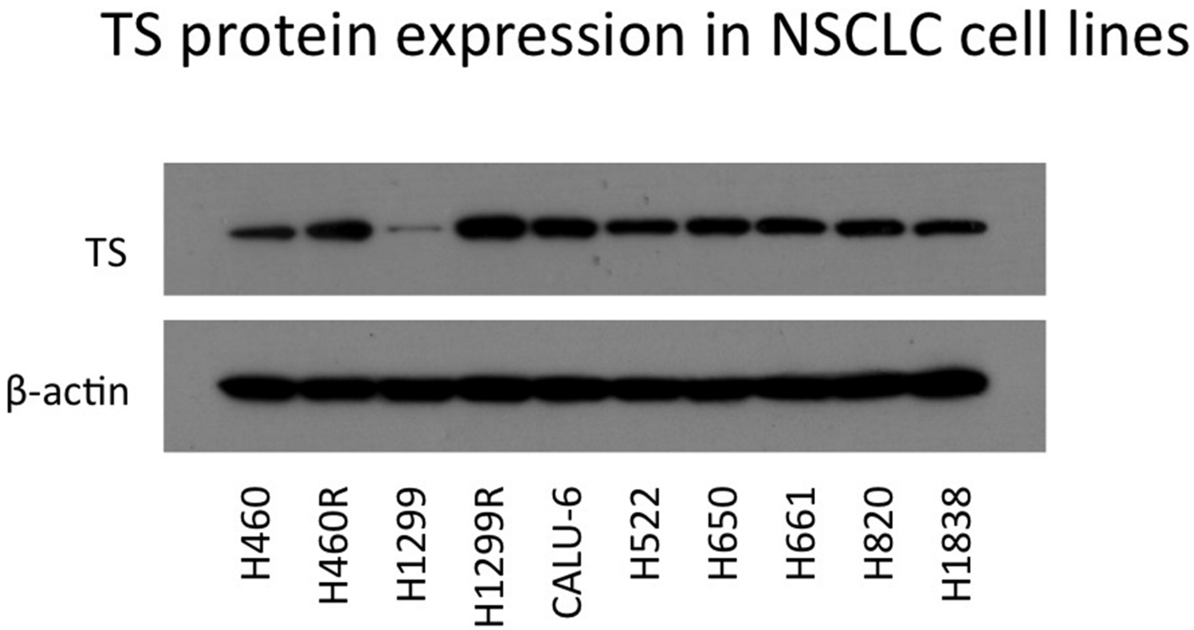

Supplementary Figure 1: Thymidylate synthase expression in NSCLC cell lines. TS protein expression was lower in the pemetrexed sensitive lines, H460 and H1299, relative to the remainder of the tested NSCLC cell lines which were resistant to pemetrexed.

NSCLC cell line sensitivity to pemetrexed

Cell lines	IC50 (µM)	Cell lines	IC50 (µM)
H460	0.141	H460R	22.8
H1299	0.656	H1299R	213
H23	0.221	CALU6	14.2
H1975	0.731	H522	59.6
H1703	0.618	H1838	121
HCC827	0.175	H661	85.0
H1650	0.182	H650	238
H1155	0.402	H820	48.0

Supplementary Figure 2: Pemetrexed sensitivity of NSCLC cell lines. The IC50 was calculated for each NSCLC cell line.

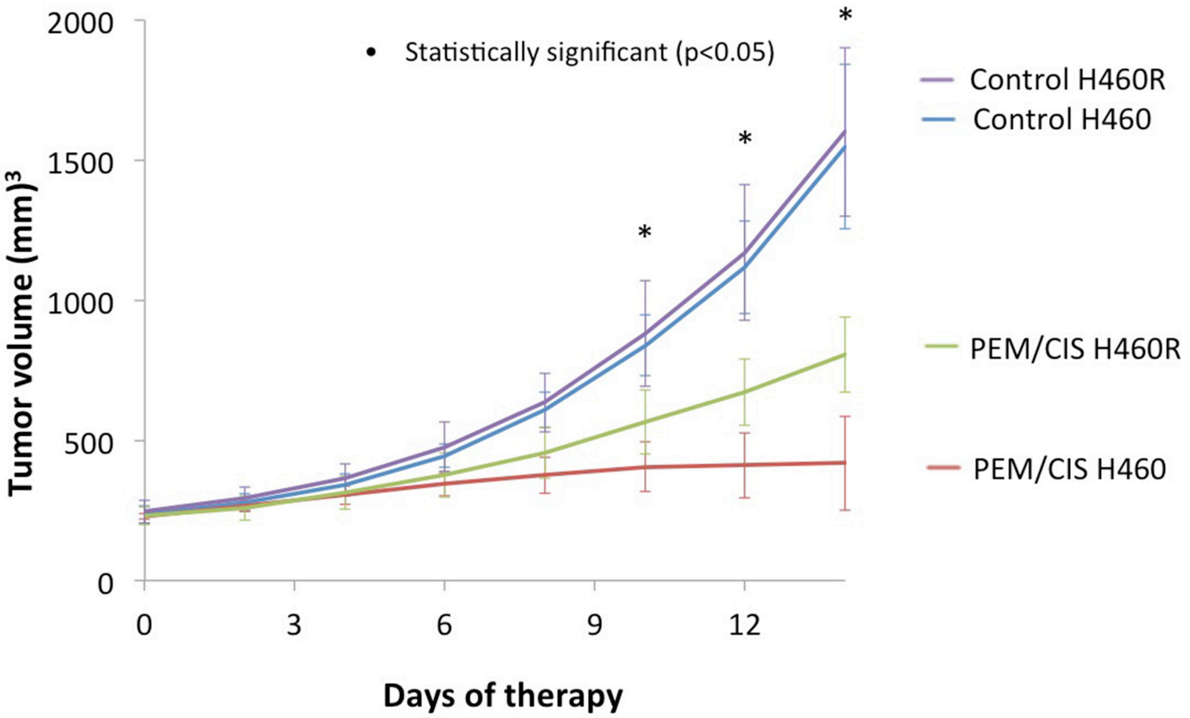

Supplementary Figure 3: Overexpression of TS results in decreased tumor growth inhibition relative to wild-type pemetrexed H460 *in vivo*. H460 and H406R bearing xenografted mice were either treated as controls or given combination therapy with cisplatin and pemetrexed (PEM/CIS). By day 14 of therapy, H460R xenografts exhibited significantly less tumor growth inhibition compared to the wild-type H460 xenografts, although both groups had significant tumor growth inhibition compared to the untreated controls.
